# Assessment of Anti m-RNA SARS-CoV-2 (BNT162b2) Antibody Titres in Mother and Child Pairs of Breastfeeding Women Vaccinated Post-Delivery

**DOI:** 10.3390/vaccines10071089

**Published:** 2022-07-07

**Authors:** Bożena Kociszewska-Najman, Magdalena Jaskólska, Karol Taradaj, Elopy Sibanda, Tomasz Ginda

**Affiliations:** 1 Department of Neonatology and Rare Diseases, Faculty of Health Sciences, Medical University of Warsaw, 02-091 Warszawa, Poland; bnajman@wum.edu.pl (B.K.-N.); karol.taradaj@wum.edu.pl (K.T.); tomasz.ginda@wum.edu.pl (T.G.); 2 Faculty of Medicine, National University of Science and Technology in Bulawayo, Bulawayo P.O. Box AC 909, Zimbabwe; ensibanda@gmail.com

**Keywords:** COVID-19, vaccination, breastfeeding, immunity, antibodies, infants

## Abstract

The global response to the COVID-19 pandemic has been successfully driven by efforts to ramp up access to vaccines. Pregnant or breastfeeding women and their children have not benefited from the vaccines despite their susceptibility to the virus. We investigated whether women who were offered vaccination after delivery passively transferred protective antibodies to their infants via breast milk. Serum was collected from breast feeding mother–infant pairs and analysed for levels of antibodies to the SARS-CoV-2 spike protein using the CLIA chemiluminescence technique. Data were analysed for the significance of the differences using the Mann–Whitney U test and the Spearman’s rank correlation coefficient to determine the strength of the correlation. A total of 13 mothers, mean age 34.86 (95%CI = 33.21–36.48) years and their infants, mean age 15.77 (95%CI = 11.24–20.29) months were enrolled. The mothers had completed their courses of the mRNA BNT162b2 SARS-CoV-2 vaccine during breastfeeding, 8.3 (95%CI = 7.24–9.36) months before the study. All 13 mothers had detectable antibodies to the SARS-CoV-2 spike protein, mean 1252, (95%CI = 736–1769) BAU/mL. Antibodies were detected in 3/13 (23%) breast-fed infants mean 322, (95%CI = 252–897) BAU/mL. There was no correlation between the maternal and infant IgG antibody titres. The time-lag since full vaccination did not correlate to the presence of antibodies in infant sera. Maternal or infant ages did not correlate to the presence of antibodies. Although some children (23%) had anti-SARS-CoV-2 antibodies, there was no association between vaccine-induced COVID-19 spike protein specific maternal IgG antibody titres and the presence of antibodies in the breastfed infants. The data show that the transfer of passive immunity to infants following post-partum vaccination with the mRNA BNT162b2 SARS-CoV-2 vaccine may be infrequent in this population.

## 1. Introduction

The SARS-CoV-2 virus is one of the greatest epidemiological challenges of the 21st century and a major cause of death in the world. The disease caused by SARS-CoV-2, COVID-19 can lead to serious complications and death. At the time of writing this article, the number of COVID-19 victims has exceeded 6 million worldwide [1]. In the initial phases of the pandemic, when the dominant SARS-CoV-2 were the Alpha and Beta variants, the vast majority of infections in the paediatric population were asymptomatic or only pauci-symptomatic. The emergence of new SARS-CoV-2 variants including the Delta and Omicron variants has dramatically changed the patient profile, causing severe infections and mortality in children as well.

In the absence of an effective causal treatment, the only documented method of prevention so far is vaccination. Whereas adults and increasingly, older children are eligible for vaccination, susceptible neonates and infants have no access to any vaccines. Currently, the European Medicines Agency (EMA) and the Food and Drugs Agency (FDA) have approved five and three products, respectively. An innovative vaccine based on mRNA technology, the BNT162b2 COVID-19 Vaccine (Pfizer–BioNTech) was used for the first time in the primary prevention of SARS-CoV-2 infections. There are no approved preparations for neonates and infants.

COVID-19 vaccines are safe and highly effective in the prevention of SARS-CoV-2 infections [2]. However, the vaccines are currently not registered by the FDA and EMA for pregnant and lactating women and infants. The databases of medical publications contain the first results of studies on the immunogenicity of COVID-19 vaccines in pregnant/breastfeeding participants, however, the sample sizes are rather small [3]. This issue requires further research on representative groups of patients.

SARS-CoV-2 infection during pregnancy is dangerous for both the mother and the foetus. The main threat of COVID-19 infection during pregnancy is preterm labour [4] and, consequently, prematurity with all its complications. Moreover, as shown by Naidu et al. [5], the cytokine storm associated with the infection may induce neurological disorders in developmental age.

The attainment of specific immunity in this group of children is currently hindered by the absence of approved paediatric vaccine formulations, and the absence of authorization to vaccinate pregnant women. The approval for infant immunization remains a remote possibility even as children increasingly succumb to the condition. Currently, the only route through which neonates and infants can access any level of COVID-19 immunity is by the passive transfer of antibodies from a vaccinated or previously infected mother. The presence of vaccine-induced antibodies in children of mothers who were vaccinated during pregnancy has been demonstrated [6].

The IgG antibodies cross the placenta and have been detected in cord blood [7,8], where their presence is presumed to confer immune protection. Transplacental transfer of IgG antibodies has been demonstrated for other vaccinations recommended during pregnancy, including those for influenza and whooping cough [9]. 

Antibodies are also present in the milk of mothers vaccinated during pregnancy or lactation. In addition to immunoglobulins, breast milk contains a number of components that support the development of the baby’s immune system and thus protects against the development of viral and bacterial infections. Substances playing a key role in protection against infections include oligosaccharides, proteins (such as lactoferrin), lipids, and pro- and anti-inflammatory factors (TNF-α, interleukin-1, interleukin-10, prostaglandins E2, etc.) [10,11]. Of particular interest is lactoferrin which acts by inhibiting the multiplication of viruses, including RSV, as demonstrated by in vitro studies [12]. Lactoferrin also inhibits infections caused by adenoviruses [12]. When discussing the positive influence of breastfeeding on the development of the child’s immunity, the presence of immune cells in the mother’s milk cannot be omitted. In the conducted study, the presence of CD45+ leukocyte populations and cells such as myeloid precursors, neutrophils, immature granulocytes, CD16+ and CD16- monocytes, non-cytotoxic T cells, cytotoxic T and NK cells, eosinophils, basophils, B-cell precursors, and B cells was proved by flow cytometry [13]. The immune cells contained in breast milk are able to survive in the gastrointestinal environment and enter the bloodstream through the mucosa, modulating the cellular response of the child’s immune system and directly phagocytosing pathogens [14,15].

Numerous studies have highlighted the role of breastfeeding in protection against infections. Christensen et al. [16] showed that breastfeeding reduces the risk of hospitalisation due to infection in the first year of a child’s life. In contrast, Verd et al. directly demonstrated that breastfed children are less likely to be infected with SARS-CoV-2 compared to children fed only milk replacer (*p* = 0.036) [17].

Immunization of pregnant women protects the pregnant woman and the foetus, as well as the new-born, and later the infant, before they can be vaccinated themselves. Immunization against SARS-CoV-2 infection has many benefits for both the mother and the neonate. Despite these advantages, knowledge about the effects of the COVID-19 vaccine on breastfeeding mothers and their babies is very limited. It is unknown whether vaccination during breastfeeding also protects the breastfed child. For this reason, many global scientific societies recommend vaccinations in this group despite the lack of registration [18,19]. Although there is abundant evidence of immune protection following the transplacental transfer of IgG antibodies, it remains unclear whether breastfeeding confers any immunological protection. 

The objective of the study is to answer the question of whether the children of mothers vaccinated against COVID-19 during breastfeeding obtain passive immunity owing to antibodies contained in the breast milk. 

## 2. Material and Methods

### 2.1. Material

The study included 13 pairs of mothers and their children. The age of women ranged from 32 to 40 years (34.84 ± 2.70 years) and children from 8 to 29 months (15.76 ± 7.49 months). A total of 4 boys (31%) and 9 girls (69%) were included in the study. During the study, the mothers were lactating. The study was not restricted exclusively to breastfeeding women. Women using different feeding patterns were qualified to reflect the lactation patterns of the study population. At the same time, none of the women included in the study had a history of documented SARS-CoV-2 infection. Detailed criteria for inclusion and exclusion are presented in Table 1.

### 2.2. Methods

The study was designed as a comparative analysis of IgG anti-SARS-CoV-2 antibody titres in mothers and infants. A survey was also conducted in which mothers were asked about lactation and social behavior during the COVID-19 epidemic. The questionnaire and the results are presented in Table 2. The study was conducted between August and September 2021 at the Department of Neonatology and Rare Diseases, Medical University of Warsaw, Poland. The study was conducted after obtaining a positive opinion from the Bioethical Committee of the Medical University of Warsaw. Venous blood samples, 3 mL from the mothers and 3 mL from the children were collected. Blood samples were centrifuged 90 min after collection in a centrifuge (centrifugation parameters: 3000 G for 10 min) to obtain serum. The serum was analysed using the CLIA chemiluminescence method, standardized against the international WHO standard NIBSC 20/136 with use of the Liaison analyser (Diasorin, Saluggia, Italy). The test determines IgG antibodies specific for the S-1 and S-2 units of the SARS-CoV-2 virus surface antigens. The result was expressed in BAU/mL. The StatSoft Statistica ver. 13.3 software was used for the statistical analysis of the results. Due to the non-fulfilment of the assumptions of the parametric tests caused by the small sample size, non-parametric tests were used in the analysis. The Mann–Whitney U test was used to assess the significance of the differences, and the strength of the correlation was determined by evaluation of the Spearman’s rank correlation coefficient.

## 3. Results

The results are presented in Table 3 The concentration of neutralizing antibodies (IgG), specific for the peak protein—S antigen below <4.81 BAU/mL, was considered a negative result, and >33.8 BAU/mL a positive result.

All tested women (n = 13) had detectable anti-SARS-CoV-2 S protein IgG antibodies. Values ranged from 614.00 to >2080, mean 1252 (95% CI = 736–1769) BAU/mL. In 7/13 (54%) of the mothers, values were greater than the upper limit of detection (>2080 BAU/mL). Three children (23%) tested positive for anti-SARS-CoV-2 S IgG antibodies. The values ranged from 4.81 to 588, mean 322, (95% CI = −253–897) BAU/mL. 

**Figure 1:**
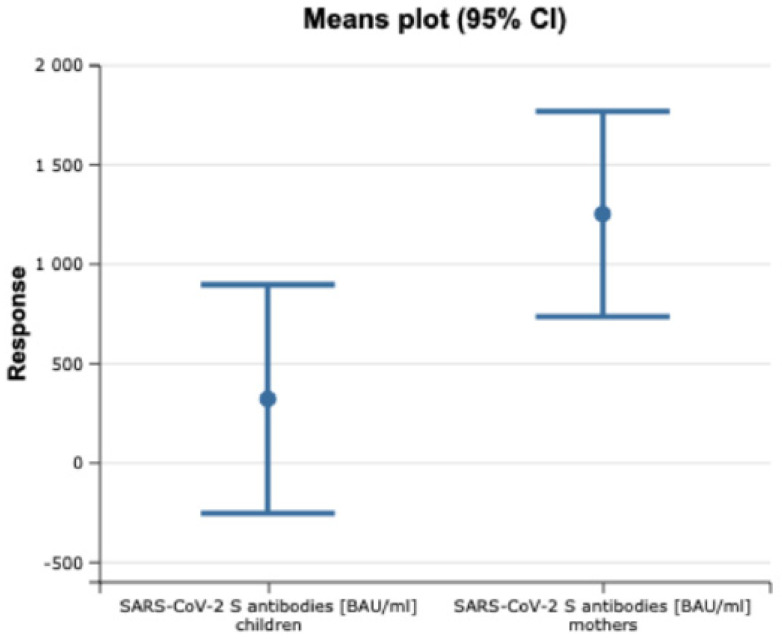
Anti-SARS-CoV-2 S protein IgG antibody titre results.

**Figure 2:**
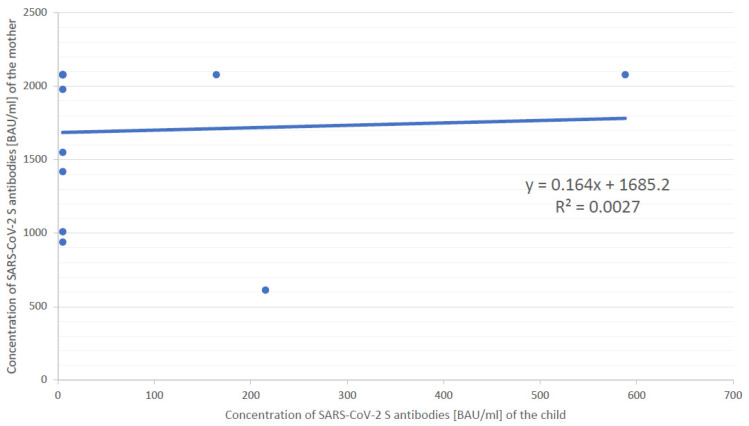
Correlation between maternal and child IgG antibody titre results. Blue dot: Concentration of SARS-CoV-2 S antibodies in specific mother—child pair.

There were differences in the levels of SARS-CoV-2 S protein IgG antibodies between the mothers and their breastfed babies. The results were statistically significant (*p* = 0.02, Mann–Whitney U test) (Figure 1). However, it was found that there was no correlation between the titre of SARS-CoV-2 S IgG antibodies in the mother and the child (R = 0.00, Spearman’s rank correlation coefficient) (Figure 2). There was a weak correlation between the time interval from vaccination and the titre of antibodies in the mother’s serum (R = −0.13, Spearman’s rank correlation coefficient). However, the results were not statistically significant (*p* > 0.05).

## 4. Discussion

Recent waves of the COVID-19 pandemic have shown that children of all ages are susceptible to infection and disease, yet there are currently no approved vaccines for these age groups. In this small study we investigated whether antibodies present in breast milk could confer passive immunity to the breastfeeding infants. Our results show that the concentrations of SARS-CoV-2 antibodies in mothers who had received two doses of the mRNA vaccine (BNT162b2) against COVID-19 after childbirth were uniformly high and, in some cases, higher than the detection limit of the kit used. The presence of significant IgG antibodies was detected in a quarter (3/13) of the paired children. The physiological mechanism of transmission of IgA and IgG antibodies to infants via breast milk is well recognized. However, the reason for the selective transfer of antibodies in the breast milk to some children is unclear. We are unaware of similar studies comparing the antibody titres in post-partum vaccinated mothers with those in their breastfed children. 

The chronological appearance of IgA and IgG antibodies in breast milk has been reported by others. According to Perl et al. [20], IgA antibodies appear in breast milk two weeks after the administration of the first vaccine dose. Titres increase significantly after receiving the second dose of the vaccine (four weeks from the beginning of the vaccination course). IgG antibodies were detected in a significant titre only after the second dose. In this study, 8.3 (7.24–9.36) months had elapsed since the administration of the second vaccine dose and breast milk IgG levels were expected to be high. However, there was no correlation between the presence of antibodies in the mother’s plasma and the child’s plasma (R = 0.00, Spearman’s rank correlation coefficient). The results of this study suggest that the presence of antibodies in maternal serum may not be related to their presence in the serum of the breastfed child. 

It should be acknowledged that the source of antibodies in the three children may not have been from the maternal breast milk. Gray et al. [7] showed the presence of SARS-CoV-2 S IgG antibodies in the cord blood of all mothers vaccinated during pregnancy, thereby demonstrating that transplacental transfer of antibodies to the foetus was probably universal. Bearing in mind that COVID-19 is a predominantly asymptomatic infection, a possibility that the three children could have acquired the antibodies transplacentally following an asymptomatic infection of their mothers cannot be ruled out. The children may also have developed antibodies following asymptomatic infection. Another variable that was not interrogated is the quantum of milk received by the antibody-positive versus antibody-negative children.

The infrequent detection of antibodies in breastfed infants compared to the universal presence in cord blood shows that the transplacental exposure to SARS-CoV-2 antibodies is superior to breast milk. These findings should help allay the vaccine hesitancy reported by Blakeway et al. [21] who followed 1328 pregnant women, with only 140 agreeing to be vaccinated during pregnancy. Younger women (<30) were less likely to be vaccinated (*p* = 0.001). Pregnant women constitute a special group in terms of introducing new drugs and pharmaceutical products including vaccines. Multiphase studies are required and, therefore, COVID-19 vaccines in this group of patients are not currently registered due to an abundance of caution rather than a lack of evidence of efficacy. Numerous studies confirm the safety of BNT162b2 vaccination for the mother and the foetus. The frequency and severity of side effects are comparable to those of the general population. According to Shimabukuro et al. [3] there were no differences in the incidence of obstetric complications between patients vaccinated and unvaccinated against COVID-19 during pregnancy. In another study Blakeway et al. [21] obtained similar results, but the results were not statistically significant (*p* > 0.05). In the case of this study [21], there was a significant disproportion in numbers between vaccinated and unvaccinated mothers during pregnancy (1188 vs. 140), which could be the reason for the lack of significant differences, while the trend was maintained. The safety of COVID-19 vaccines in pregnant women was also confirmed by Ciapponi et al. [22], who extensively reviewed medical databases, involving 2,398,855 pregnant women in 38 studies, 37 of which reported no significant undesirable obstetric complications after vaccination performed during pregnancy.

We have demonstrated the coexistence of antibodies in three mother–child pairs. The origin of this correlation cannot be unequivocally explained by the fact that the child received antibodies from the mother’s milk, as the history of asymptomatic COVID-19 infection cannot be ruled out. The study was conducted among children over 6 months of age whose diet contains food other than breast milk. 

Based on the questionnaire (the form and results are presented in Table 3), an interview with the mothers was conducted regarding breastfeeding and social behavior during the COVID-19 pandemic (during the period in which a blood sample was taken for anti-SARS-CoV-2 antibody testing). The aim of the questionnaire study was to objectify the obtained results of immunological tests. Despite mothers taking measures to limit the transmission of the virus (limiting interpersonal contacts, working remotely), ultimately all children with positive antibody titres came into contact with households infected with COVID-19. There may be no connection between immunizing a lactating mother and immunizing her baby. Presumably, the presence of antibodies in children is the result of children’s contact with infected people.

There is insufficient data and studies on a large group of children clearly confirming the effectiveness of protection of children by immunization of breastfeeding mothers. The variable concentration of antibodies in mothers is also interesting.

In summary, it should be emphasized that women vaccinated with both doses of BNT162b2 vaccine during breastfeeding benefit from immunization and produce adequate SARS-CoV-2 S IgG antibodies. This benefit does not seem to be shared with their breastfed children with only a quarter having detectable antibodies. The findings contrast starkly with those observed in mothers vaccinated during pregnancy wherein antibodies were detected in all cord blood samples. However, immunization during pregnancy has not been widely recommended and is associated with considerable hesitancy. Clinicians’ attention should be drawn to the role of COVID-19 immunization education in pregnant women. Immunization during pregnancy should be promoted in order to obtain immunity in newborns and infants for whom immunization is currently not recommended. Immunization should be carried out in the second and third trimesters of pregnancy after the organogenesis processes are completed.

## 5. Conclusions

1. The breastfeeding women vaccinated with BNT162b2 demonstrate anti-SARS-CoV-2 antibodies between 6 and 10 weeks after vaccination.
2. It cannot be concluded from this study that breastfeeding by women vaccinated against COVID-19 during lactation does not lead to a passive immune response in their children. Confirmation of the conclusion requires studies on a larger population with a uniform feeding schedule, including in particular exclusively breastfed children.

## Figures and Tables

**Table 1:** Inclusion and exclusion criteria.

Inclusion Criteria	Exclusion Criteria
Age from 18 to 45	18 < age < 45
Taking two doses of BNT162b2 (Comirnaty, Pfizer–BioNTech) vaccine after childbirth	Vaccination before childbirth
Negative history of SARS-CoV-2 infection prior to mother or child vaccination	Failure to complete the full vaccination schedule with BNT162b2 (Comirnaty, Pfizer–BioNTech)
Time from taking the second dose of the vaccine is 6 to 12 weeks	Time from the second dose of vaccine less than 6 or more than 12 weeks
No immunodeficiency	SARS-CoV-2 infection confirmed by PCR or antigen test before immunization of the mother or child
Single pregnancy	Congenital or acquired immunodeficiencies
Childbirth after 37 weeks of pregnancy	Use of immunosuppressive drugs
Physiological delivery and caesarean section	Multiple pregnancy
Active lactation and breastfeeding	Birth defects
Informed consent to participate in the study	Premature delivery <38 weeks of pregnancy
	Lack of informed consent to participate in the study

**Table 2:** Results of a survey on lactation and social behaviour.

	Frequency (n)/%	Including the Mother of the Child with Positive IgG Antibodies (n)/%
How often have you been breastfeeding?		
*a. Main feeding*	1 (8%)	0 (0%)
*b. Several times a day: during the day and at night*	9 (69%)	2 (15%)
*c. Twice/ three times per day*	3 (23%)	0 (0%)
*d. Sporadically*	0 (0%)	0 (0%)
Are you employed?		
*a. Yes*	1 (8%)	0 (0%)
*b. Yes, I work online*	7 (54%)	2 (15%)
*c. No*	5 (38%)	1 (8%)
Was any close family contact with suspected or diagnosed with COVID-19 person after childbirth?		
*a. No*	4 (31%)	2 (15%)
*b. Yes*	9 (69%)	1 (8%)
Did the household members was diagnosed for COVID-19?		
*a. Yes*	3 (23%)	3 (23%)
*b. No*	10 (77%)	0 (0%)

**Table 3:** Concentration of SARS-CoV-2 S IgG antibodies in the serum of mothers and their children.

Concentration of SARS-CoV-2 S Antibodies [BAU/mL] of the Mother (Mean 1252 (95% CI = 736–1769) BAU/mL)	Mother’s Age [Years] (34.84 ± 2.70 Years)	Time from the 2nd Dose of Vaccination to Blood Collection [Weeks]	Concentration of SARS-CoV-2 S Antibodies [BAU/mL] of the Child (Mean 322, (95% CI = −253–897) BAU/mL)	Child’s Age [Months] (Mean 15.76 ± 7.49 Months)	Child’s Gender (31% Male 69% Female)
1420	32	6	<4.81	14	female
614	33	7	215.00	28	female
>2080	35	7	<4.81	13	male
1010	40	7	<4.81	23	female
>2080	32	7	<4.81	11	female
>2080	35	12	<4.81	10	male
>2080	35	8	<4.81	29	male
940	36	9	<4.81	24	female
1550	33	7	<4.81	10	male
1980	32	10	<4.81	15	female
>2080	36	8	<4.81	8	female
>2080	34	10	164.00	10	female
>2080	40	10	588.00	10	female

## Data Availability

Not applicable.

## References

[1] WHO COVID-19 Dashboard. https://covid19.who.int/.

[2] Polack F.P., Thomas S.J., Kitchin N., Absalon J., Gurtman A., Lockhart S., Perez J.L., Pérez Marc G., Moreira E.D., Zerbini C. (2020). Safety and efficacy of the BNT162b2 mRNA COVID-19 vaccine. N. Engl. J. Med..

[3] Shimabukuro T.T., Kim S.Y., Myers T.R., Moro P.L., Oduyebo T., Panagiotakopoulos L., Marquez P.L., Olson C.K., Liu R., Chang K.T. (2021). Preliminary Findings of mRNA COVID-19 Vaccine Safety in Pregnant Persons. N. Engl. J. Med..

[4] Adhikari E.H., Spong C.Y. (2021). COVID-19 Vaccination in Pregnant and Lactating Women. JAMA.

[5] Naidu M.S.A.G., Clemens D.R.A., Pressman P., Zaigham B.M., Kadkhoda K., Davies K.J.A., Naidu A.S. (2022). COVID-19 during Pregnancy and Postpartum. J. Diet. Suppl..

[6] Halasa N.B., Olson S.M., Staat M.A., Newhams M.M., Price A.M., Boom J.A., Sahni L.C., Cameron M.A., Pannaraj P.S., Bline K.E. (2022). Effectiveness of Maternal Vaccination with mRNA COVID-19 Vaccine During Pregnancy Against COVID-19–Associated Hospitalization in Infants Aged <6 Months—17 States, July 2021–January 2022. MMWR. Morb. Mortal. Wkly. Rep..

[7] Gray K.J., Bordt E.A., Atyeo C., Deriso E., Akinwunmi B., Young N., Edlow A.G. (2021). COVID-19 vaccine response in pregnant and lactating women: A cohort study. medRxiv.

[8] Falsaperla R., Leone G., Familiari M., Ruggieri M. (2021). COVID-19 vaccination in pregnant and lactating women: A systematic review. Expert Rev. Vaccines.

[9] Maertens K., Orije M.R.P., Van Damme P., Leuridan E. (2020). Vaccination during pregnancy: Current and possible future recommendations. Eur. J. Pediatr..

[10] Bränn E., Edvinsson Å., Punga A.R., Sundström-Poromaa I., Skalkidou A. (2019). Inflammatory and anti-inflammatory markers in plasma: From late pregnancy to early postpartum. Sci. Rep..

[11] Lang J., Yang N., Deng J., Liu K., Yang P., Zhang G., Jiang C. (2011). Inhibition of SARS Pseudovirus Cell Entry by Lactoferrin Binding to Heparan Sulfate Proteoglycans. PLoS ONE.

[12] Wakabayashi H., Oda H., Yamauchi K., Abe F. (2014). Lactoferrin for prevention of common viral infections. J. Infect. Chemother..

[13] Trend S., de Jong E., Lloyd M.L., Kok C.H., Richmond P., Doherty D.A., Simmer K., Kakulas F., Strunk T., Currie A. (2015). Leukocyte Populations in Human Preterm and Term Breast Milk Identified by Multicolour Flow Cytometry. PLoS ONE.

[14] Witkowska-Zimny M., Kaminska-El-Hassan E. (2017). Cells of human breast milk. Cell. Mol. Biol. Lett..

[15] Hassiotou F., Geddes D.T. (2015). Immune Cell–Mediated Protection of the Mammary Gland and the Infant during Breastfeeding. Adv. Nutr. Int. Rev. J..

[16] Christensen N., Bruun S., Søndergaard J., Christesen H.T., Fisker N., Zachariassen G., Sangild P.T., Husby S. (2020). Breastfeeding and Infections in Early Childhood: A Cohort Study. Pediatrics.

[17] Verd S., Ramakers J., Vinuela I., Martin-Delgado M.-I., Prohens A., Díez R. (2021). Does breastfeeding protect children from COVID-19? An observational study from pediatric services in Majorca, Spain. Int. Breastfeed. J..

[18] Luxi N., Giovanazzi A., Capuano A., Crisafulli S., Cutroneo P.M., Fantini M.P., Ferrajolo C., Moretti U., Poluzzi E., Raschi E. (2021). COVID-19 Vaccination in Pregnancy, Paediatrics, Immunocompromised Patients, and Persons with History of Allergy or Prior SARS-CoV-2 Infection: Overview of Current Recommendations and Pre- and Post-Marketing Evidence for Vaccine Efficacy and Safety. Drug Saf..

[19] Nassar A.H., Visser G.H.A., Nicholson W.K., Ramasauskaite D., Kim Y.H., Barnea E.R., Escobar M.F., Pacagnella R., Theron G., Wright A. (2021). FIGO Statement: Vaccination in pregnancy. Int. J. Gynecol. Obstet..

[20] Perl S.H., Uzan-Yulzari A., Klainer H., Asiskovich L., Youngster M., Rinott E., Youngster I. (2021). SARS-CoV-2–Specific Antibodies in Breast Milk After COVID-19 Vaccination of Breastfeeding Women. JAMA.

[21] Blakeway H., Prasad S., Kalafat E., Heath P.T., Ladhani S.N., Le Doare K., Magee L.A., O’Brien P., Rezvani A., von Dadelszen P. (2022). COVID-19 vaccination during pregnancy: Coverage and safety. Am. J. Obstet. Gynecol..

[22] Ciapponi A., Bardach A., Mazzoni A., Alconada T., Anderson A.S., Argento F.J., Ballivian J., Bok K., Comandé D., Erbelding E. (2021). Safety of components and platforms of COVID-19 vaccines considered for use in pregnancy: A rapid review. Vaccine.

